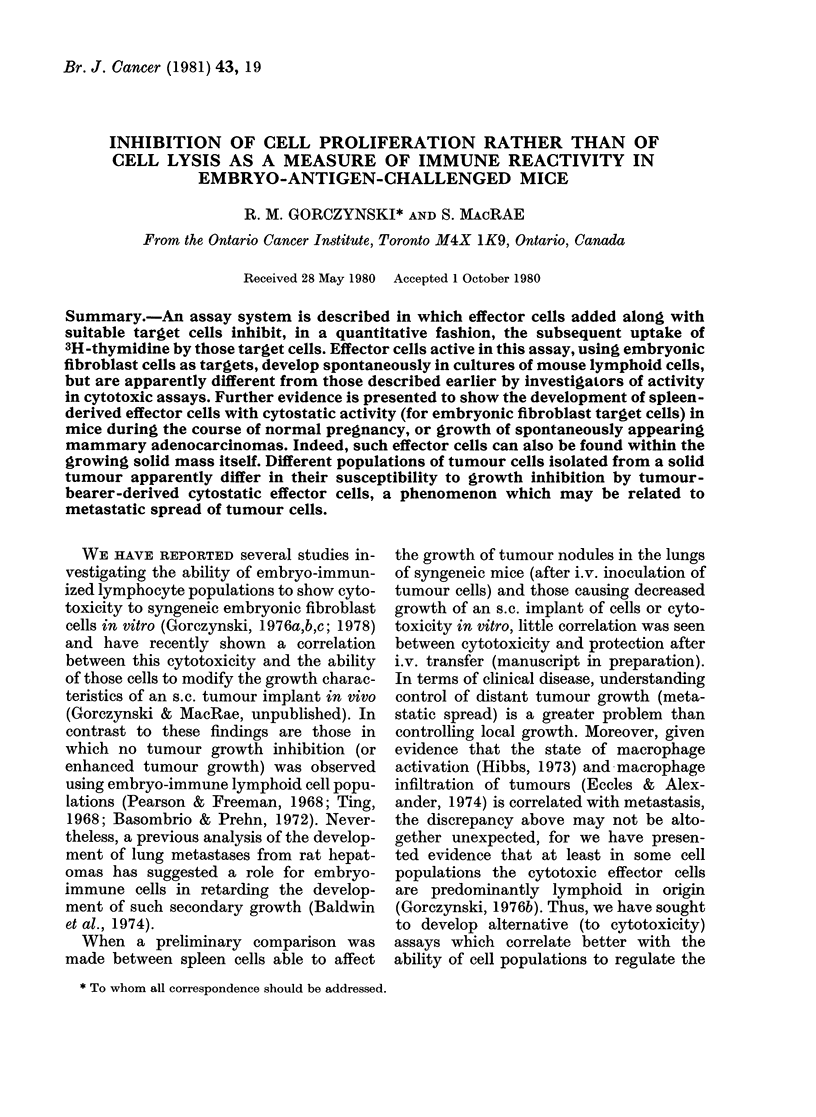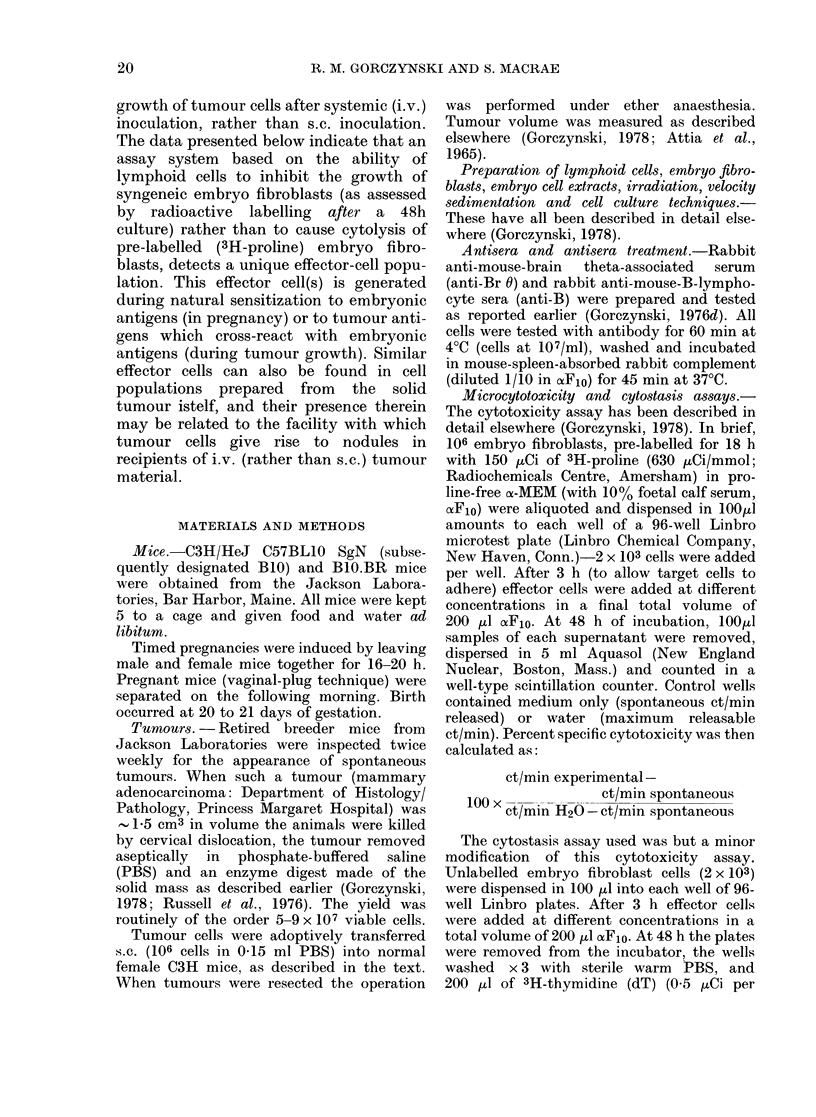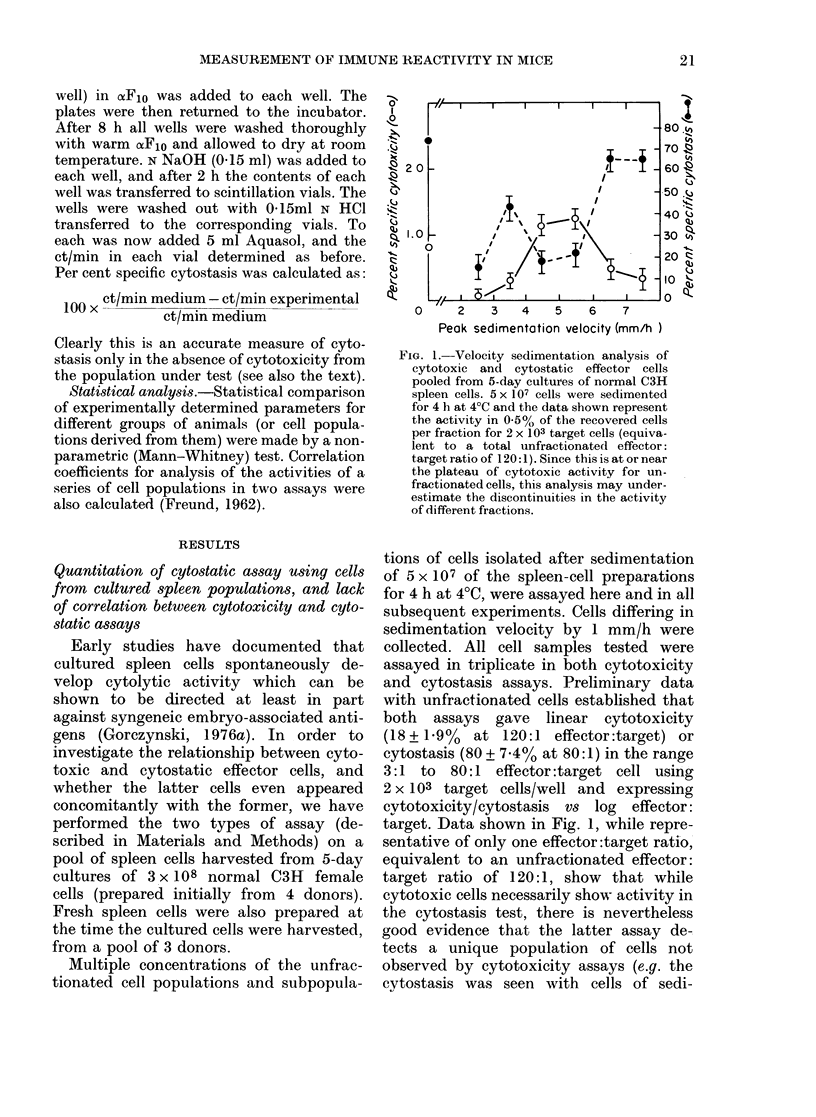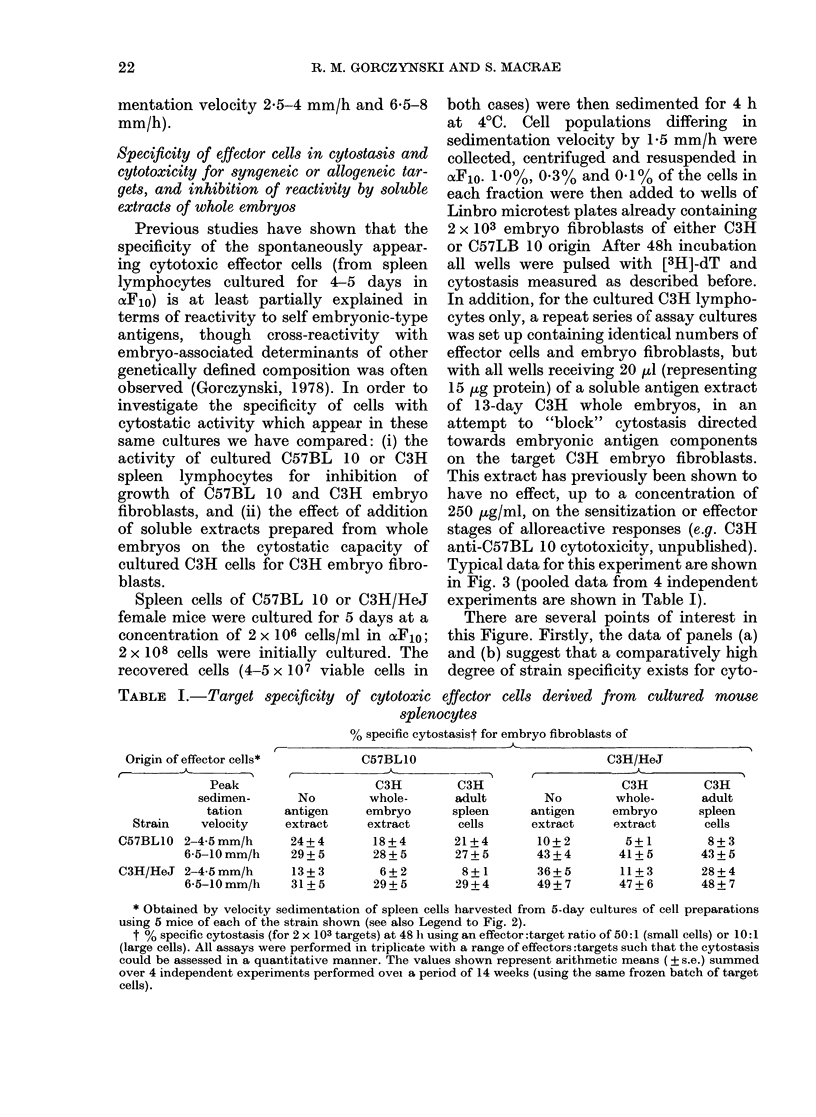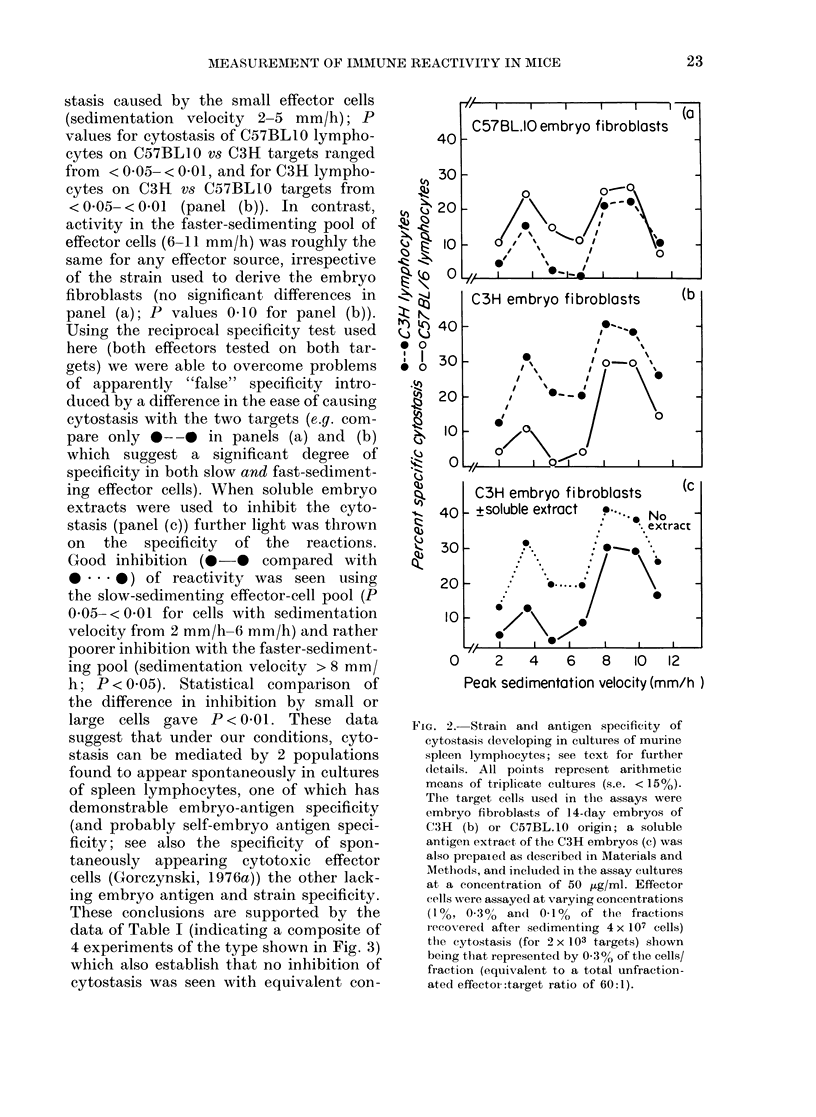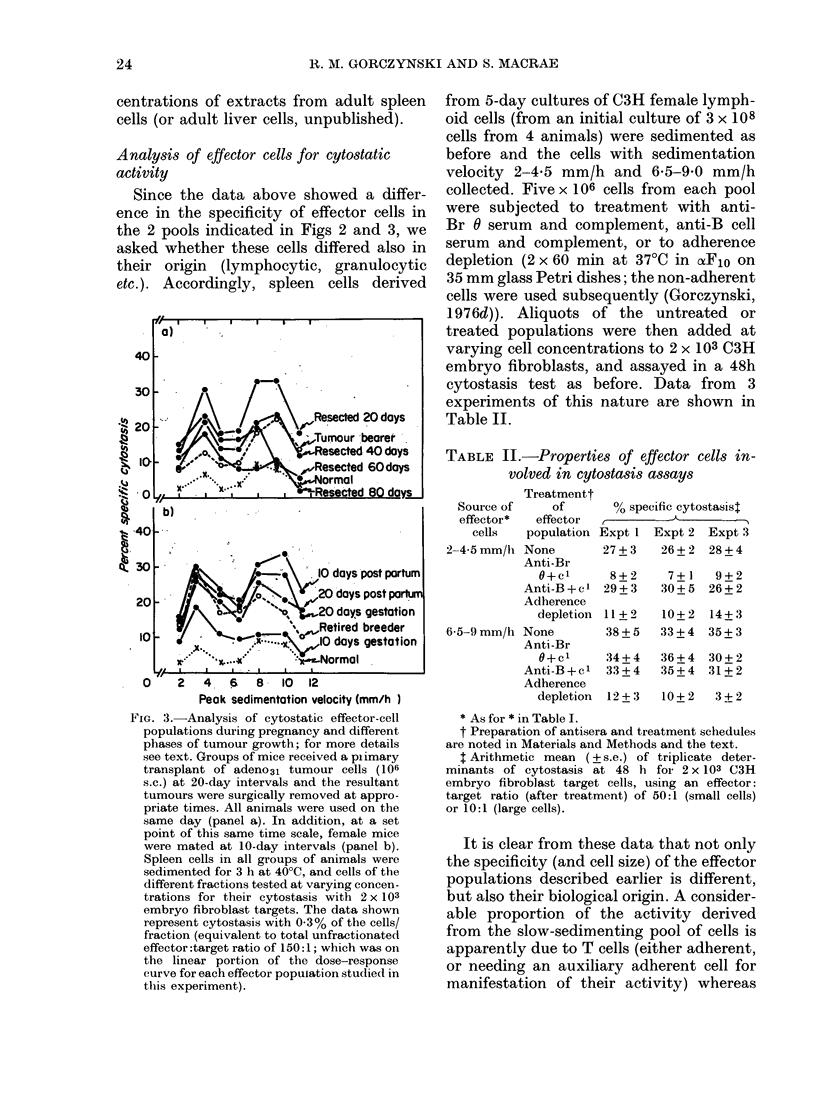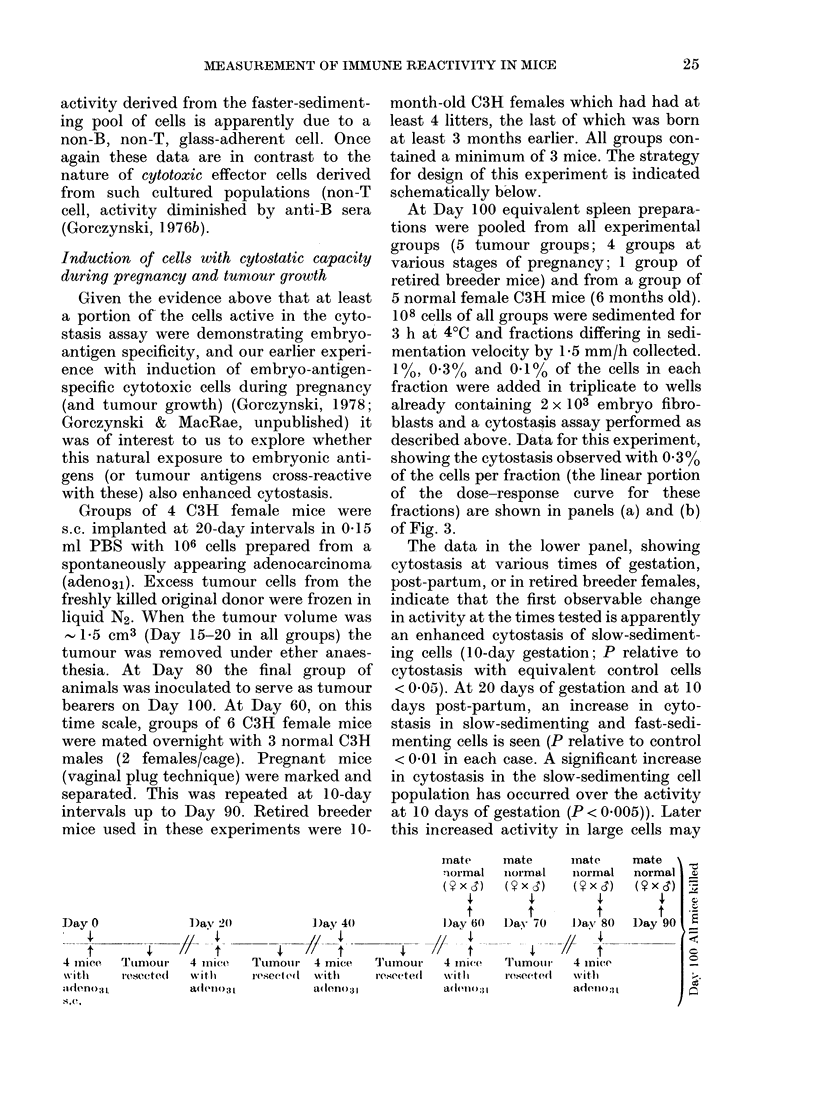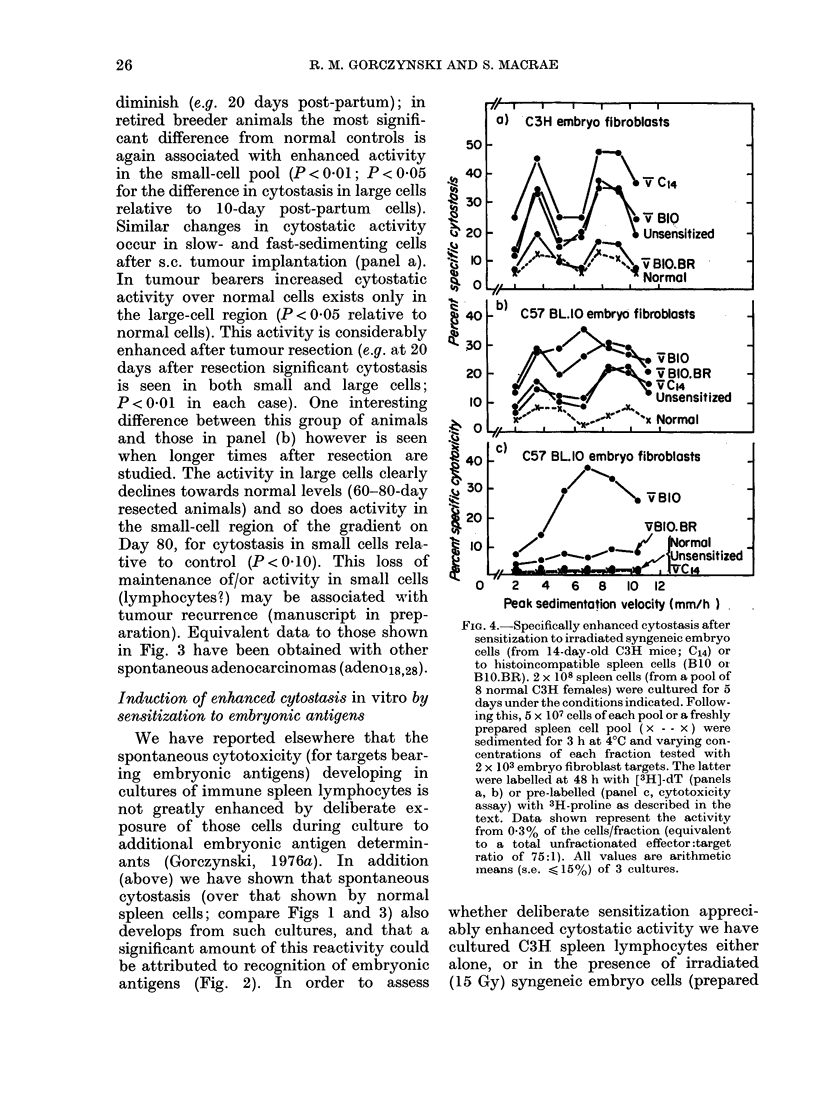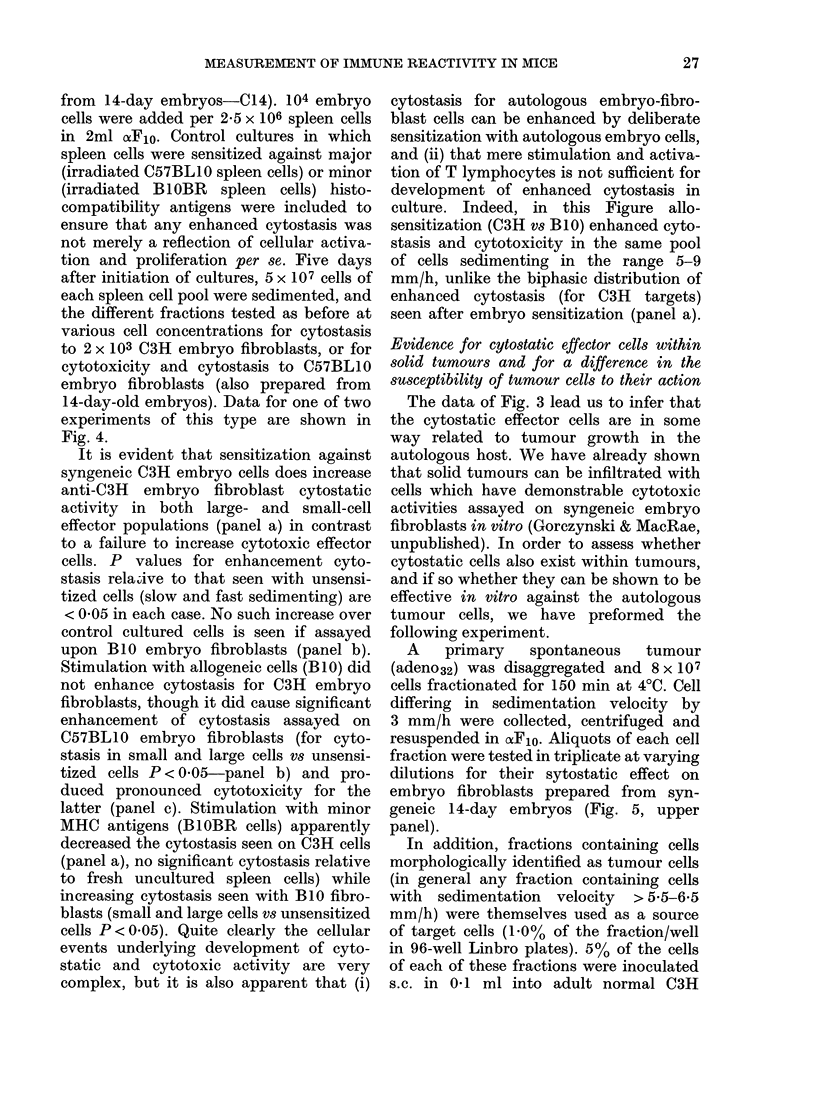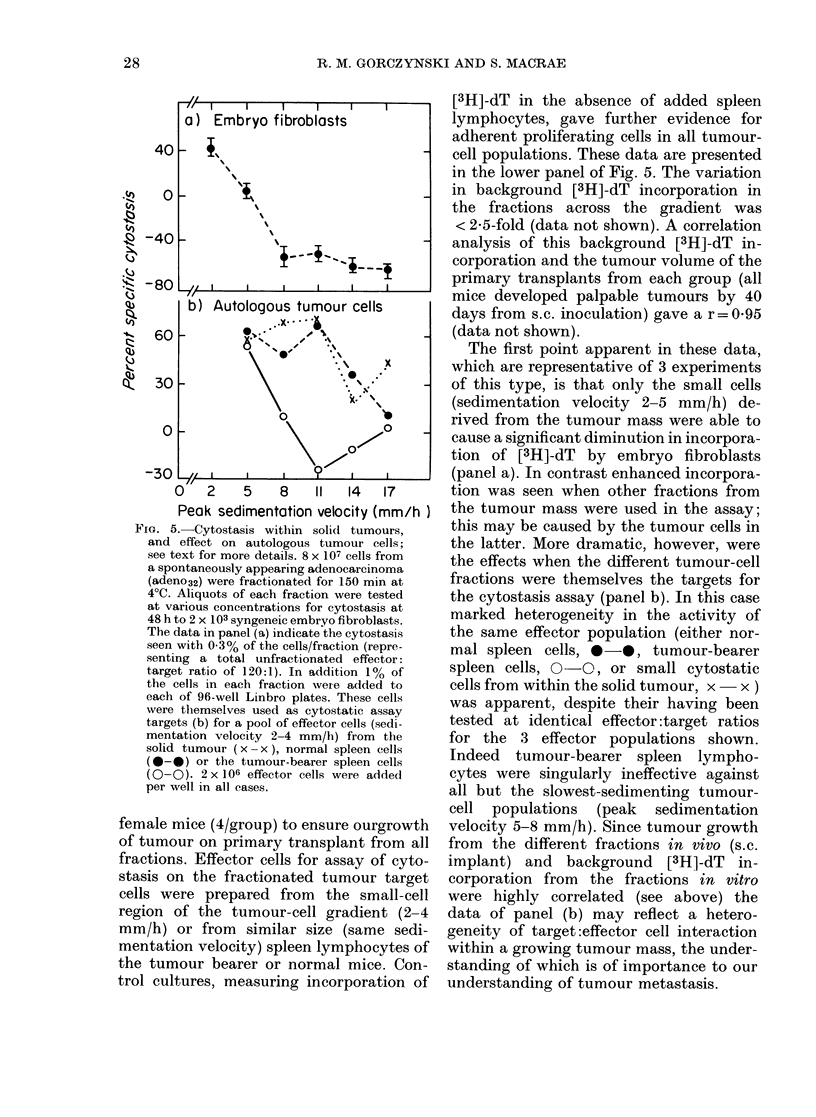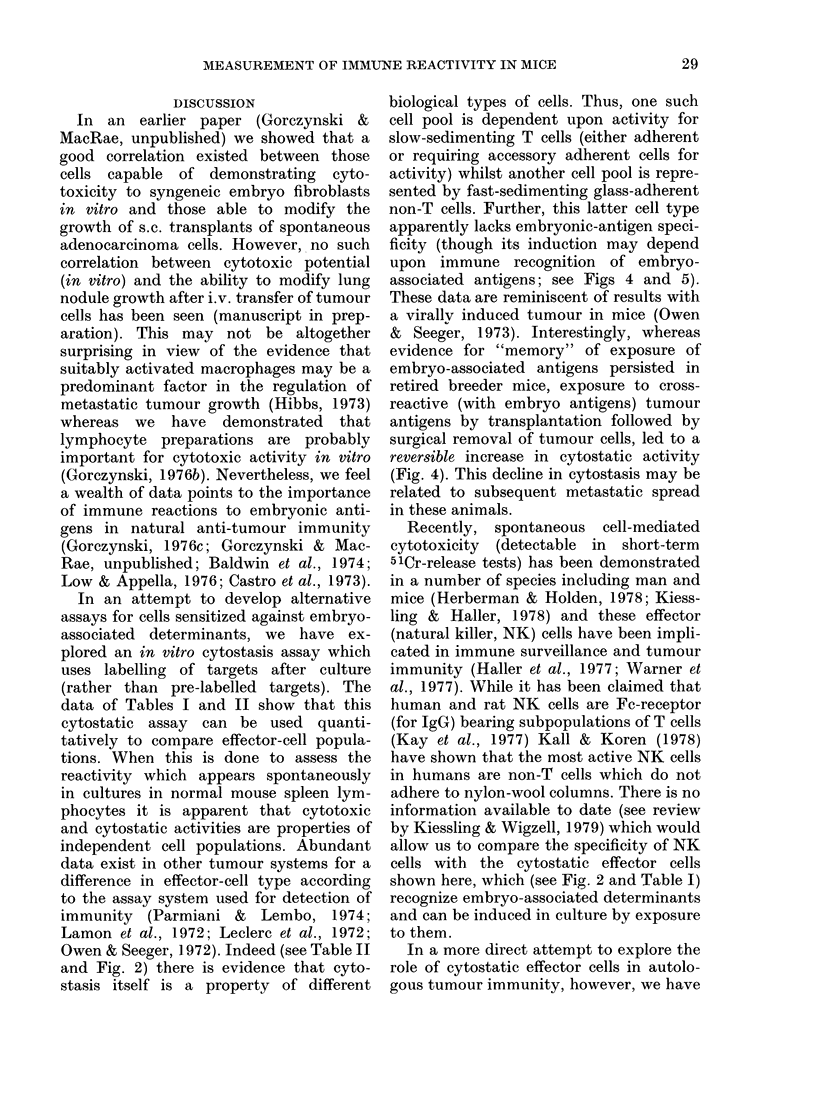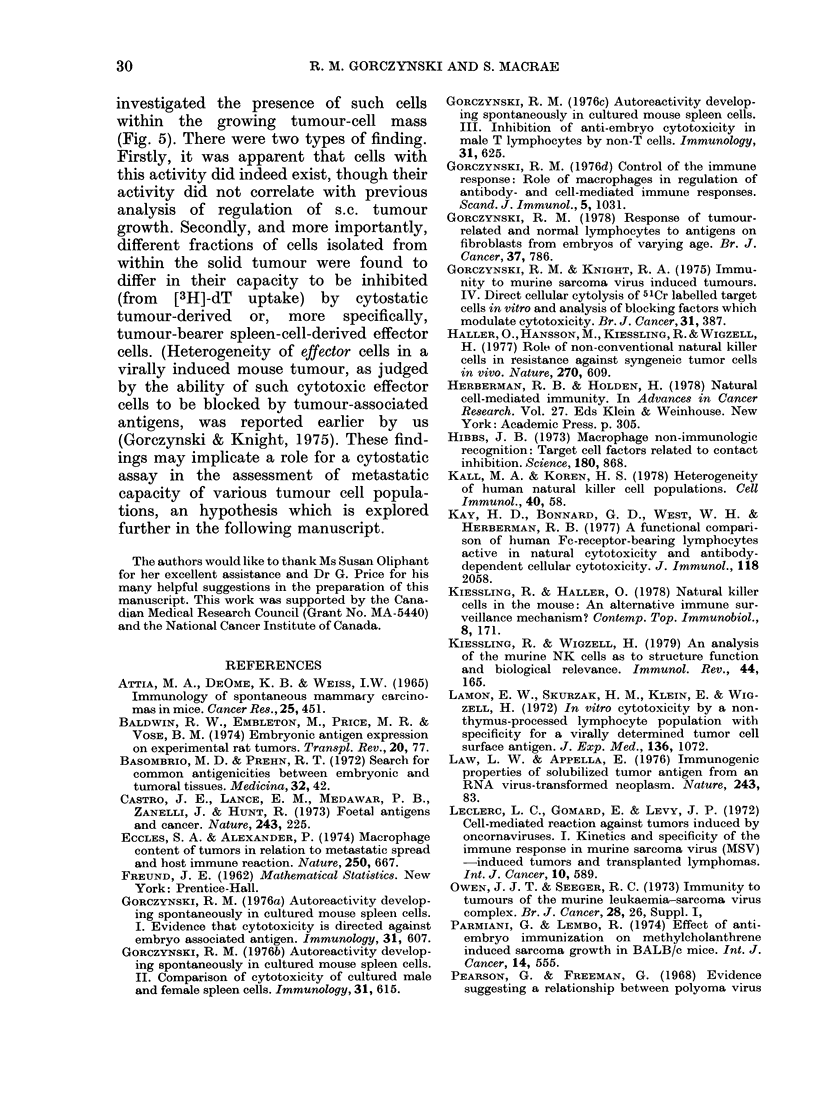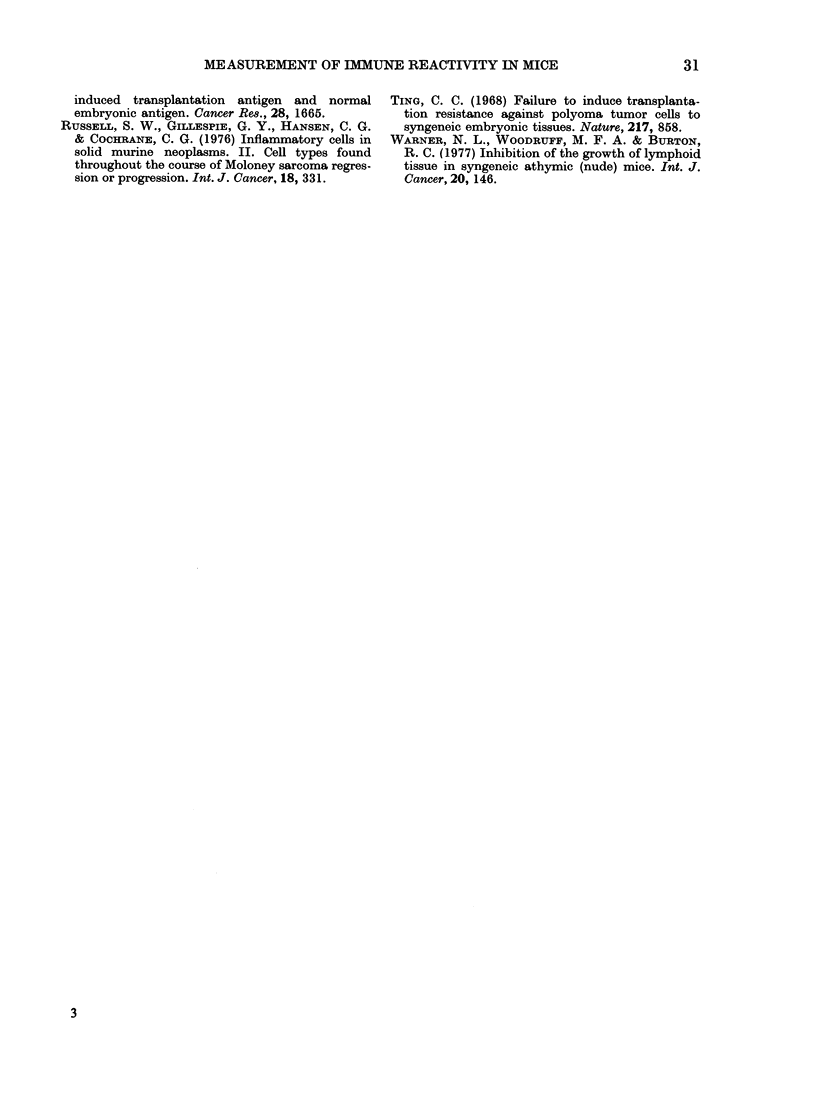# Inhibition of cell proliferation rather than of cell lysis as a measure of immune reactivity in embryo-antigen-challenged mice.

**DOI:** 10.1038/bjc.1981.4

**Published:** 1981-01

**Authors:** R. M. Gorczynski, S. MacRae

## Abstract

An assay system is described in which effector cells added along with suitable target cells inhibit, in a quantitative fashion, the subsequent uptake of 3H-thymidine by those target cells. Effector cells active in this assay, using embryonic fibroblast cells as targets, develop spontaneously in cultures of mouse lymphoid cells, but are apparently different from those described earlier by investigators of activity in cytotoxic assays. Further evidence is presented to show the development of spleen-derived effector cells with cytostatic activity (for embryonic fibroblast target cells) in mice during the course of normal pregnancy, or growth of spontaneously appearing mammary adenocarcinomas. Indeed, such effector cells can also be found within the growing solid mass itself. Different populations of tumour cells isolated from a solid tumour apparently differ in their susceptibility to growth inhibition by tumour-bearer-derived cytostatic effector cells, a phenomenon which may be related to metastatic spread of tumour cells.


*Br. J. Cancer* (1981) **43**, 19

### INHIBITION OF CELL PROLIFERATION RATHER THAN OF CELL LYSIS AS A MEASURE OF IMMUNE REACTIVITY IN EMBRYO-ANTIGEN-CHALLENGED MICE

R. M. GORCZYNSKI* AND S. MACRAE

*From the Ontario Cancer Institute, Toronto M4X 1K9, Ontario, Canada*

Received 28 May 1980 Accepted 1 October 1980

**Summary.—An assay system is described in which effector cells added along with suitable target cells inhibit, in a quantitative fashion, the subsequent uptake of $^3$H-thymidine by those target cells. Effector cells active in this assay, using embryonic fibroblast cells as targets, develop spontaneously in cultures of mouse lymphoid cells, but are apparently different from those described earlier by investigators of activity in cytotoxic assays. Further evidence is presented to show the development of spleen-derived effector cells with cytostatic activity (for embryonic fibroblast target cells) in mice during the course of normal pregnancy, or growth of spontaneously appearing mammary adenocarcinomas. Indeed, such effector cells can also be found within the growing solid mass itself. Different populations of tumour cells isolated from a solid tumour apparently differ in their susceptibility to growth inhibition by tumour-bearer-derived cytostatic effector cells, a phenomenon which may be related to metastatic spread of tumour cells.**

WE HAVE REPORTED several studies investigating the ability of embryo-immunized lymphocyte populations to show cytotoxicity to syngeneic embryonic fibroblast cells *in vitro* (Gorczynski, 1976*a,b,c*; 1978) and have recently shown a correlation between this cytotoxicity and the ability of those cells to modify the growth characteristics of an s.c. tumour implant *in vivo* (Gorczynski & MacRae, unpublished). In contrast to these findings are those in which no tumour growth inhibition (or enhanced tumour growth) was observed using embryo-immune lymphoid cell populations (Pearson & Freeman, 1968; Ting, 1968; Basombrio & Prehn, 1972). Nevertheless, a previous analysis of the development of lung metastases from rat hepatomas has suggested a role for embryo-immune cells in retarding the development of such secondary growth (Baldwin *et al.*, 1974).

When a preliminary comparison was made between spleen cells able to affect the growth of tumour nodules in the lungs of syngeneic mice (after i.v. inoculation of tumour cells) and those causing decreased growth of an s.c. implant of cells or cytotoxicity *in vitro*, little correlation was seen between cytotoxicity and protection after i.v. transfer (manuscript in preparation). In terms of clinical disease, understanding control of distant tumour growth (metastatic spread) is a greater problem than controlling local growth. Moreover, given evidence that the state of macrophage activation (Hibbs, 1973) and macrophage infiltration of tumours (Eccles & Alexander, 1974) is correlated with metastasis, the discrepancy above may not be altogether unexpected, for we have presented evidence that at least in some cell populations the cytotoxic effector cells are predominantly lymphoid in origin (Gorczynski, 1976*b*). Thus, we have sought to develop alternative (to cytotoxicity) assays which correlate better with the ability of cell populations to regulate the

* To whom all correspondence should be addressed.

growth of tumour cells after systemic (i.v.) inoculation, rather than s.c. inoculation. The data presented below indicate that an assay system based on the ability of lymphoid cells to inhibit the growth of syngeneic embryo fibroblasts (as assessed by radioactive labelling *after* a 48h culture) rather than to cause cytolysis of pre-labelled ($^3$H-proline) embryo fibroblasts, detects a unique effector-cell population. This effector cell(s) is generated during natural sensitization to embryonic antigens (in pregnancy) or to tumour antigens which cross-react with embryonic antigens (during tumour growth). Similar effector cells can also be found in cell populations prepared from the solid tumour istelf, and their presence therein may be related to the facility with which tumour cells give rise to nodules in recipients of i.v. (rather than s.c.) tumour material.

### MATERIALS AND METHODS

*Mice.*—C3H/HeJ C57BL10 SgN (subsequently designated B10) and B10.BR mice were obtained from the Jackson Laboratories, Bar Harbor, Maine. All mice were kept 5 to a cage and given food and water *ad libitum.*

Timed pregnancies were induced by leaving male and female mice together for 16–20 h. Pregnant mice (vaginal-plug technique) were separated on the following morning. Birth occurred at 20 to 21 days of gestation.

*Tumours.* — Retired breeder mice from Jackson Laboratories were inspected twice weekly for the appearance of spontaneous tumours. When such a tumour (mammary adenocarcinoma: Department of Histology/Pathology, Princess Margaret Hospital) was $\sim 1{\cdot}5$ cm$^3$ in volume the animals were killed by cervical dislocation, the tumour removed aseptically in phosphate-buffered saline (PBS) and an enzyme digest made of the solid mass as described earlier (Gorczynski, 1978; Russell *et al.*, 1976). The yield was routinely of the order $5–9 \times 10^7$ viable cells.

Tumour cells were adoptively transferred s.c. ($10^6$ cells in 0·15 ml PBS) into normal female C3H mice, as described in the text. When tumours were resected the operation was performed under ether anaesthesia. Tumour volume was measured as described elsewhere (Gorczynski, 1978; Attia *et al.*, 1965).

*Preparation of lymphoid cells, embryo fibroblasts, embryo cell extracts, irradiation, velocity sedimentation and cell culture techniques.*—These have all been described in detail elsewhere (Gorczynski, 1978).

*Antisera and antisera treatment.*—Rabbit anti-mouse-brain theta-associated serum (anti-Br $\theta$) and rabbit anti-mouse-B-lymphocyte sera (anti-B) were prepared and tested as reported earlier (Gorczynski, 1976*d*). All cells were tested with antibody for 60 min at 4°C (cells at $10^7$/ml), washed and incubated in mouse-spleen-absorbed rabbit complement (diluted 1/10 in $\alpha F_{10}$) for 45 min at 37°C.

*Microcytotoxicity and cytostasis assays.*—The cytotoxicity assay has been described in detail elsewhere (Gorczynski, 1978). In brief, $10^6$ embryo fibroblasts, pre-labelled for 18 h with 150 $\mu$Ci of $^3$H-proline (630 $\mu$Ci/mmol; Radiochemicals Centre, Amersham) in proline-free $\alpha$-MEM (with 10% foetal calf serum, $\alpha F_{10}$) were aliquoted and dispensed in 100$\mu$l amounts to each well of a 96-well Linbro microtest plate (Linbro Chemical Company, New Haven, Conn.)—$2 \times 10^3$ cells were added per well. After 3 h (to allow target cells to adhere) effector cells were added at different concentrations in a final total volume of 200 $\mu$l $\alpha F_{10}$. At 48 h of incubation, 100$\mu$l samples of each supernatant were removed, dispersed in 5 ml Aquasol (New England Nuclear, Boston, Mass.) and counted in a well-type scintillation counter. Control wells contained medium only (spontaneous ct/min released) or water (maximum releasable ct/min). Percent specific cytotoxicity was then calculated as:

$$100 \times \frac{\text{ct/min experimental} - \text{ct/min spontaneous}}{\text{ct/min } H_2O - \text{ct/min spontaneous}}$$

The cytostasis assay used was but a minor modification of this cytotoxicity assay. Unlabelled embryo fibroblast cells ($2 \times 10^3$) were dispensed in 100 $\mu$l into each well of 96-well Linbro plates. After 3 h effector cells were added at different concentrations in a total volume of 200 $\mu$l $\alpha F_{10}$. At 48 h the plates were removed from the incubator, the wells washed $\times 3$ with sterile warm PBS, and 200 $\mu$l of $^3$H-thymidine (dT) (0·5 $\mu$Ci per well) in $\alpha F_{10}$ was added to each well. The plates were then returned to the incubator. After 8 h all wells were washed thoroughly with warm $\alpha F_{10}$ and allowed to dry at room temperature. N NaOH (0·15 ml) was added to each well, and after 2 h the contents of each well was transferred to scintillation vials. The wells were washed out with 0·15ml N HCl transferred to the corresponding vials. To each was now added 5 ml Aquasol, and the ct/min in each vial determined as before. Per cent specific cytostasis was calculated as:

$$100 \times \frac{\text{ct/min medium} - \text{ct/min experimental}}{\text{ct/min medium}}$$

Clearly this is an accurate measure of cytostasis only in the absence of cytotoxicity from the population under test (see also the text).

*Statistical analysis.*—Statistical comparison of experimentally determined parameters for different groups of animals (or cell populations derived from them) were made by a non-parametric (Mann–Whitney) test. Correlation coefficients for analysis of the activities of a series of cell populations in two assays were also calculated (Freund, 1962).

FIG. 1.—Velocity sedimentation analysis of cytotoxic and cytostatic effector cells pooled from 5-day cultures of normal C3H spleen cells. $5 \times 10^7$ cells were sedimented for 4 h at 4°C and the data shown represent the activity in 0·5% of the recovered cells per fraction for $2 \times 10^3$ target cells (equivalent to a total unfractionated effector: target ratio of 120:1). Since this is at or near the plateau of cytotoxic activity for unfractionated cells, this analysis may underestimate the discontinuities in the activity of different fractions.

### RESULTS

### *Quantitation of cytostatic assay using cells from cultured spleen populations, and lack of correlation between cytotoxicity and cytostatic assays*

Early studies have documented that cultured spleen cells spontaneously develop cytolytic activity which can be shown to be directed at least in part against syngeneic embryo-associated antigens (Gorczynski, 1976*a*). In order to investigate the relationship between cytotoxic and cytostatic effector cells, and whether the latter cells even appeared concomitantly with the former, we have performed the two types of assay (described in Materials and Methods) on a pool of spleen cells harvested from 5-day cultures of $3 \times 10^8$ normal C3H female cells (prepared initially from 4 donors). Fresh spleen cells were also prepared at the time the cultured cells were harvested, from a pool of 3 donors.

Multiple concentrations of the unfractionated cell populations and subpopulations of cells isolated after sedimentation of $5 \times 10^7$ of the spleen-cell preparations for 4 h at 4°C, were assayed here and in all subsequent experiments. Cells differing in sedimentation velocity by 1 mm/h were collected. All cell samples tested were assayed in triplicate in both cytotoxicity and cytostasis assays. Preliminary data with unfractionated cells established that both assays gave linear cytotoxicity ($18 \pm 1{\cdot}9\%$ at 120:1 effector:target) or cytostasis ($80 \pm 7{\cdot}4\%$ at 80:1) in the range 3:1 to 80:1 effector:target cell using $2 \times 10^3$ target cells/well and expressing cytotoxicity/cytostasis *vs* log effector: target. Data shown in Fig. 1, while representative of only one effector:target ratio, equivalent to an unfractionated effector: target ratio of 120:1, show that while cytotoxic cells necessarily show activity in the cytostasis test, there is nevertheless good evidence that the latter assay detects a unique population of cells not observed by cytotoxicity assays (*e.g.* the cytostasis was seen with cells of sedimentation velocity 2·5–4 mm/h and 6·5–8 mm/h).

*Specificity of effector cells in cytostasis and cytotoxicity for syngeneic or allogeneic targets, and inhibition of reactivity by soluble extracts of whole embryos*

Previous studies have shown that the specificity of the spontaneously appearing cytotoxic effector cells (from spleen lymphocytes cultured for 4–5 days in $\alpha F_{10}$) is at least partially explained in terms of reactivity to self embryonic-type antigens, though cross-reactivity with embryo-associated determinants of other genetically defined composition was often observed (Gorczynski, 1978). In order to investigate the specificity of cells with cytostatic activity which appear in these same cultures we have compared: (i) the activity of cultured C57BL 10 or C3H spleen lymphocytes for inhibition of growth of C57BL 10 and C3H embryo fibroblasts, and (ii) the effect of addition of soluble extracts prepared from whole embryos on the cytostatic capacity of cultured C3H cells for C3H embryo fibroblasts.

Spleen cells of C57BL 10 or C3H/HeJ female mice were cultured for 5 days at a concentration of $2\times10^6$ cells/ml in $\alpha F_{10}$; $2\times10^8$ cells were initially cultured. The recovered cells ($4$–$5\times10^7$ viable cells in both cases) were then sedimented for 4 h at 4°C. Cell populations differing in sedimentation velocity by 1·5 mm/h were collected, centrifuged and resuspended in $\alpha F_{10}$. 1·0%, 0·3% and 0·1% of the cells in each fraction were then added to wells of Linbro microtest plates already containing $2\times10^3$ embryo fibroblasts of either C3H or C57LB 10 origin After 48h incubation all wells were pulsed with [$^3$H]-dT and cytostasis measured as described before. In addition, for the cultured C3H lymphocytes only, a repeat series of assay cultures was set up containing identical numbers of effector cells and embryo fibroblasts, but with all wells receiving 20 $\mu$l (representing 15 $\mu$g protein) of a soluble antigen extract of 13-day C3H whole embryos, in an attempt to "block" cytostasis directed towards embryonic antigen components on the target C3H embryo fibroblasts. This extract has previously been shown to have no effect, up to a concentration of 250 $\mu$g/ml, on the sensitization or effector stages of alloreactive responses (*e.g.* C3H anti-C57BL 10 cytotoxicity, unpublished). Typical data for this experiment are shown in Fig. 3 (pooled data from 4 independent experiments are shown in Table I).

There are several points of interest in this Figure. Firstly, the data of panels (a) and (b) suggest that a comparatively high degree of strain specificity exists for cyto-

TABLE I.—*Target specificity of cytotoxic effector cells derived from cultured mouse splenocytes*

| Origin of effector cells* | | % specific cytostasis† for embryo fibroblasts of | | | | | |
|---|---|---|---|---|---|---|---|
| | | C57BL10 | | | C3H/HeJ | | |
| Strain | Peak sedimentation velocity | No antigen extract | C3H whole-embryo extract | C3H adult spleen cells | No antigen extract | C3H whole-embryo extract | C3H adult spleen cells |
| C57BL10 | 2–4·5 mm/h | 24 ± 4 | 18 ± 4 | 21 ± 4 | 10 ± 2 | 5 ± 1 | 8 ± 3 |
| | 6·5–10 mm/h | 29 ± 5 | 28 ± 5 | 27 ± 5 | 43 ± 4 | 41 ± 5 | 43 ± 5 |
| C3H/HeJ | 2–4·5 mm/h | 13 ± 3 | 6 ± 2 | 8 ± 1 | 36 ± 5 | 11 ± 3 | 28 ± 4 |
| | 6·5–10 mm/h | 31 ± 5 | 29 ± 5 | 29 ± 4 | 49 ± 7 | 47 ± 6 | 48 ± 7 |

* Obtained by velocity sedimentation of spleen cells harvested from 5-day cultures of cell preparations using 5 mice of each of the strain shown (see also Legend to Fig. 2).

† % specific cytostasis (for $2\times10^3$ targets) at 48 h using an effector:target ratio of 50:1 (small cells) or 10:1 (large cells). All assays were performed in triplicate with a range of effectors:targets such that the cytostasis could be assessed in a quantitative manner. The values shown represent arithmetic means (±s.e.) summed over 4 independent experiments performed ovei a period of 14 weeks (using the same frozen batch of target cells).

stasis caused by the small effector cells (sedimentation velocity 2–5 mm/h); $P$ values for cytostasis of C57BL10 lymphocytes on C57BL10 *vs* C3H targets ranged from $< 0{\cdot}05$–$< 0{\cdot}01$, and for C3H lymphocytes on C3H *vs* C57BL10 targets from $< 0{\cdot}05$–$< 0{\cdot}01$ (panel (b)). In contrast, activity in the faster-sedimenting pool of effector cells (6–11 mm/h) was roughly the same for any effector source, irrespective of the strain used to derive the embryo fibroblasts (no significant differences in panel (a); $P$ values 0·10 for panel (b)). Using the reciprocal specificity test used here (both effectors tested on both targets) we were able to overcome problems of apparently "false" specificity introduced by a difference in the ease of causing cytostasis with the two targets (*e.g.* compare only ●– –● in panels (a) and (b) which suggest a significant degree of specificity in both slow *and* fast-sedimenting effector cells). When soluble embryo extracts were used to inhibit the cytostasis (panel (c)) further light was thrown on the specificity of the reactions. Good inhibition (●—● compared with ● · · · ●) of reactivity was seen using the slow-sedimenting effector-cell pool ($P$ 0·05–$< 0{\cdot}01$ for cells with sedimentation velocity from 2 mm/h–6 mm/h) and rather poorer inhibition with the faster-sedimenting pool (sedimentation velocity $> 8$ mm/h; $P < 0{\cdot}05$). Statistical comparison of the difference in inhibition by small or large cells gave $P < 0{\cdot}01$. These data suggest that under our conditions, cytostasis can be mediated by 2 populations found to appear spontaneously in cultures of spleen lymphocytes, one of which has demonstrable embryo-antigen specificity (and probably self-embryo antigen specificity; see also the specificity of spontaneously appearing cytotoxic effector cells (Gorczynski, 1976*a*)) the other lacking embryo antigen and strain specificity. These conclusions are supported by the data of Table I (indicating a composite of 4 experiments of the type shown in Fig. 3) which also establish that no inhibition of cytostasis was seen with equivalent con-

FIG. 2.—Strain and antigen specificity of cytostasis developing in cultures of murine spleen lymphocytes; see text for further details. All points represent arithmetic means of triplicate cultures (s.e. $< 15\%$). The target cells used in the assays were embryo fibroblasts of 14-day embryos of C3H (b) or C57BL.10 origin; a soluble antigen extract of the C3H embryos (c) was also prepaıed as described in Materials and Methods, and included in the assay cultures at a concentration of 50 μg/ml. Effector cells were assayed at varying concentrations (1%, 0·3% and 0·1% of the fractions recovered after sedimenting $4 \times 10^7$ cells) the cytostasis (for $2 \times 10^3$ targets) shown being that represented by 0·3% of the cells/fraction (equivalent to a total unfractionated effector:target ratio of 60:1).

centrations of extracts from adult spleen cells (or adult liver cells, unpublished).

*Analysis of effector cells for cytostatic activity*

Since the data above showed a difference in the specificity of effector cells in the 2 pools indicated in Figs 2 and 3, we asked whether these cells differed also in their origin (lymphocytic, granulocytic *etc.*). Accordingly, spleen cells derived from 5-day cultures of C3H female lymphoid cells (from an initial culture of $3 \times 10^8$ cells from 4 animals) were sedimented as before and the cells with sedimentation velocity 2–4·5 mm/h and 6·5–9·0 mm/h collected. Five $\times 10^6$ cells from each pool were subjected to treatment with anti-Br $\theta$ serum and complement, anti-B cell serum and complement, or to adherence depletion ($2 \times 60$ min at 37°C in $\alpha F_{10}$ on 35 mm glass Petri dishes; the non-adherent cells were used subsequently (Gorczynski, 1976*d*)). Aliquots of the untreated or treated populations were then added at varying cell concentrations to $2 \times 10^3$ C3H embryo fibroblasts, and assayed in a 48h cytostasis test as before. Data from 3 experiments of this nature are shown in Table II.

FIG. 3.—Analysis of cytostatic effector-cell populations during pregnancy and different phases of tumour growth; for more details see text. Groups of mice received a primary transplant of adeno31 tumour cells ($10^6$ s.c.) at 20-day intervals and the resultant tumours were surgically removed at appropriate times. All animals were used on the same day (panel a). In addition, at a set point of this same time scale, female mice were mated at 10-day intervals (panel b). Spleen cells in all groups of animals were sedimented for 3 h at 40°C, and cells of the different fractions tested at varying concentrations for their cytostasis with $2 \times 10^3$ embryo fibroblast targets. The data shown represent cytostasis with 0·3% of the cells/fraction (equivalent to total unfractionated effector:target ratio of 150:1; which was on the linear portion of the dose–response curve for each effector population studied in this experiment).

TABLE II.—*Properties of effector cells involved in cytostasis assays*

| Source of effector* cells | Treatment† of effector population | % specific cytostasis‡ Expt 1 | Expt 2 | Expt 3 |
|---|---|---|---|---|
| 2–4·5 mm/h | None | 27 ± 3 | 26 ± 2 | 28 ± 4 |
| | Anti-Br $\theta$ + c[1] | 8 ± 2 | 7 ± 1 | 9 ± 2 |
| | Anti-B + c[1] | 29 ± 3 | 30 ± 5 | 26 ± 2 |
| | Adherence depletion | 11 ± 2 | 10 ± 2 | 14 ± 3 |
| 6·5–9 mm/h | None | 38 ± 5 | 33 ± 4 | 35 ± 3 |
| | Anti-Br $\theta$ + c[1] | 34 ± 4 | 36 ± 4 | 30 ± 2 |
| | Anti-B + c[1] | 33 ± 4 | 35 ± 4 | 31 ± 2 |
| | Adherence depletion | 12 ± 3 | 10 ± 2 | 3 ± 2 |

* As for * in Table I.

† Preparation of antisera and treatment schedules are noted in Materials and Methods and the text.

‡ Arithmetic mean (± s.e.) of triplicate determinants of cytostasis at 48 h for $2 \times 10^3$ C3H embryo fibroblast target cells, using an effector: target ratio (after treatment) of 50:1 (small cells) or 10:1 (large cells).

It is clear from these data that not only the specificity (and cell size) of the effector populations described earlier is different, but also their biological origin. A considerable proportion of the activity derived from the slow-sedimenting pool of cells is apparently due to T cells (either adherent, or needing an auxiliary adherent cell for manifestation of their activity) whereas activity derived from the faster-sedimenting pool of cells is apparently due to a non-B, non-T, glass-adherent cell. Once again these data are in contrast to the nature of *cytotoxic* effector cells derived from such cultured populations (non-T cell, activity diminished by anti-B sera (Gorczynski, 1976*b*).

*Induction of cells with cytostatic capacity during pregnancy and tumour growth*

Given the evidence above that at least a portion of the cells active in the cytostasis assay were demonstrating embryo-antigen specificity, and our earlier experience with induction of embryo-antigen-specific cytotoxic cells during pregnancy (and tumour growth) (Gorczynski, 1978; Gorczynski & MacRae, unpublished) it was of interest to us to explore whether this natural exposure to embryonic antigens (or tumour antigens cross-reactive with these) also enhanced cytostasis.

Groups of 4 C3H female mice were s.c. implanted at 20-day intervals in 0·15 ml PBS with $10^6$ cells prepared from a spontaneously appearing adenocarcinoma (adeno$_{31}$). Excess tumour cells from the freshly killed original donor were frozen in liquid $N_2$. When the tumour volume was $\sim 1{\cdot}5$ cm$^3$ (Day 15–20 in all groups) the tumour was removed under ether anaesthesia. At Day 80 the final group of animals was inoculated to serve as tumour bearers on Day 100. At Day 60, on this time scale, groups of 6 C3H female mice were mated overnight with 3 normal C3H males (2 females/cage). Pregnant mice (vaginal plug technique) were marked and separated. This was repeated at 10-day intervals up to Day 90. Retired breeder mice used in these experiments were 10-month-old C3H females which had had at least 4 litters, the last of which was born at least 3 months earlier. All groups contained a minimum of 3 mice. The strategy for design of this experiment is indicated schematically below.

At Day 100 equivalent spleen preparations were pooled from all experimental groups (5 tumour groups; 4 groups at various stages of pregnancy; 1 group of retired breeder mice) and from a group of 5 normal female C3H mice (6 months old). $10^8$ cells of all groups were sedimented for 3 h at 4°C and fractions differing in sedimentation velocity by 1·5 mm/h collected. 1%, 0·3% and 0·1% of the cells in each fraction were added in triplicate to wells already containing $2 \times 10^3$ embryo fibroblasts and a cytostasis assay performed as described above. Data for this experiment, showing the cytostasis observed with 0·3% of the cells per fraction (the linear portion of the dose–response curve for these fractions) are shown in panels (a) and (b) of Fig. 3.

The data in the lower panel, showing cytostasis at various times of gestation, post-partum, or in retired breeder females, indicate that the first observable change in activity at the times tested is apparently an enhanced cytostasis of slow-sedimenting cells (10-day gestation; $P$ relative to cytostasis with equivalent control cells $< 0{\cdot}05$). At 20 days of gestation and at 10 days post-partum, an increase in cytostasis in slow-sedimenting and fast-sedimenting cells is seen ($P$ relative to control $< 0{\cdot}01$ in each case. A significant increase in cytostasis in the slow-sedimenting cell population has occurred over the activity at 10 days of gestation ($P < 0{\cdot}005$)). Later this increased activity in large cells may diminish (*e.g.* 20 days post-partum); in retired breeder animals the most significant difference from normal controls is again associated with enhanced activity in the small-cell pool ($P < 0{\cdot}01$; $P < 0{\cdot}05$ for the difference in cytostasis in large cells relative to 10-day post-partum cells). Similar changes in cytostatic activity occur in slow- and fast-sedimenting cells after s.c. tumour implantation (panel a). In tumour bearers increased cytostatic activity over normal cells exists only in the large-cell region ($P < 0{\cdot}05$ relative to normal cells). This activity is considerably enhanced after tumour resection (*e.g.* at 20 days after resection significant cytostasis is seen in both small and large cells; $P < 0{\cdot}01$ in each case). One interesting difference between this group of animals and those in panel (b) however is seen when longer times after resection are studied. The activity in large cells clearly declines towards normal levels (60–80-day resected animals) and so does activity in the small-cell region of the gradient on Day 80, for cytostasis in small cells relative to control ($P < 0{\cdot}10$). This loss of maintenance of/or activity in small cells (lymphocytes?) may be associated with tumour recurrence (manuscript in preparation). Equivalent data to those shown in Fig. 3 have been obtained with other spontaneous adenocarcinomas (adeno$_{18,28}$).

*Induction of enhanced cytostasis* in vitro *by sensitization to embryonic antigens*

We have reported elsewhere that the spontaneous cytotoxicity (for targets bearing embryonic antigens) developing in cultures of immune spleen lymphocytes is not greatly enhanced by deliberate exposure of those cells during culture to additional embryonic antigen determinants (Gorczynski, 1976*a*). In addition (above) we have shown that spontaneous cytostasis (over that shown by normal spleen cells; compare Figs 1 and 3) also develops from such cultures, and that a significant amount of this reactivity could be attributed to recognition of embryonic antigens (Fig. 2). In order to assess

FIG. 4.—Specifically enhanced cytostasis after sensitization to irradiated syngeneic embryo cells (from 14-day-old C3H mice; $C_{14}$) or to histoincompatible spleen cells (B10 or B10.BR). $2 \times 10^8$ spleen cells (from a pool of 8 normal C3H females) were cultured for 5 days under the conditions indicated. Following this, $5 \times 10^7$ cells of each pool or a freshly prepared spleen cell pool (× - - ×) were sedimented for 3 h at 4°C and varying concentrations of each fraction tested with $2 \times 10^3$ embryo fibroblast targets. The latter were labelled at 48 h with [$^3$H]-dT (panels a, b) or pre-labelled (panel c, cytotoxicity assay) with $^3$H-proline as described in the text. Data shown represent the activity from 0·3% of the cells/fraction (equivalent to a total unfractionated effector:target ratio of 75:1). All values are arithmetic means (s.e. $\leqslant 15\%$) of 3 cultures.

whether deliberate sensitization appreciably enhanced cytostatic activity we have cultured C3H spleen lymphocytes either alone, or in the presence of irradiated (15 Gy) syngeneic embryo cells (prepared from 14-day embryos—C14). $10^4$ embryo cells were added per $2 \cdot 5 \times 10^6$ spleen cells in 2ml $\alpha F_{10}$. Control cultures in which spleen cells were sensitized against major (irradiated C57BL10 spleen cells) or minor (irradiated B10BR spleen cells) histocompatibility antigens were included to ensure that any enhanced cytostasis was not merely a reflection of cellular activation and proliferation *per se*. Five days after initiation of cultures, $5 \times 10^7$ cells of each spleen cell pool were sedimented, and the different fractions tested as before at various cell concentrations for cytostasis to $2 \times 10^3$ C3H embryo fibroblasts, or for cytotoxicity and cytostasis to C57BL10 embryo fibroblasts (also prepared from 14-day-old embryos). Data for one of two experiments of this type are shown in Fig. 4.

It is evident that sensitization against syngeneic C3H embryo cells does increase anti-C3H embryo fibroblast cytostatic activity in both large- and small-cell effector populations (panel a) in contrast to a failure to increase cytotoxic effector cells. $P$ values for enhancement cytostasis relative to that seen with unsensitized cells (slow and fast sedimenting) are $< 0 \cdot 05$ in each case. No such increase over control cultured cells is seen if assayed upon B10 embryo fibroblasts (panel b). Stimulation with allogeneic cells (B10) did not enhance cytostasis for C3H embryo fibroblasts, though it did cause significant enhancement of cytostasis assayed on C57BL10 embryo fibroblasts (for cytostasis in small and large cells *vs* unsensitized cells $P < 0 \cdot 05$—panel b) and produced pronounced cytotoxicity for the latter (panel c). Stimulation with minor MHC antigens (B10BR cells) apparently decreased the cytostasis seen on C3H cells (panel a), no significant cytostasis relative to fresh uncultured spleen cells) while increasing cytostasis seen with B10 fibroblasts (small and large cells *vs* unsensitized cells $P < 0 \cdot 05$). Quite clearly the cellular events underlying development of cytostatic and cytotoxic activity are very complex, but it is also apparent that (i) cytostasis for autologous embryo-fibroblast cells can be enhanced by deliberate sensitization with autologous embryo cells, and (ii) that mere stimulation and activation of T lymphocytes is not sufficient for development of enhanced cytostasis in culture. Indeed, in this Figure allosensitization (C3H *vs* B10) enhanced cytostasis and cytotoxicity in the same pool of cells sedimenting in the range 5–9 mm/h, unlike the biphasic distribution of enhanced cytostasis (for C3H targets) seen after embryo sensitization (panel a).

*Evidence for cytostatic effector cells within solid tumours and for a difference in the susceptibility of tumour cells to their action*

The data of Fig. 3 lead us to infer that the cytostatic effector cells are in some way related to tumour growth in the autologous host. We have already shown that solid tumours can be infiltrated with cells which have demonstrable cytotoxic activities assayed on syngeneic embryo fibroblasts *in vitro* (Gorczynski & MacRae, unpublished). In order to assess whether cytostatic cells also exist within tumours, and if so whether they can be shown to be effective *in vitro* against the autologous tumour cells, we have preformed the following experiment.

A primary spontaneous tumour ($adeno_{32}$) was disaggregated and $8 \times 10^7$ cells fractionated for 150 min at 4°C. Cell differing in sedimentation velocity by 3 mm/h were collected, centrifuged and resuspended in $\alpha F_{10}$. Aliquots of each cell fraction were tested in triplicate at varying dilutions for their sytostatic effect on embryo fibroblasts prepared from syngeneic 14-day embryos (Fig. 5, upper panel).

In addition, fractions containing cells morphologically identified as tumour cells (in general any fraction containing cells with sedimentation velocity $> 5 \cdot 5–6 \cdot 5$ mm/h) were themselves used as a source of target cells (1·0% of the fraction/well in 96-well Linbro plates). 5% of the cells of each of these fractions were inoculated s.c. in 0·1 ml into adult normal C3H

FIG. 5.—Cytostasis within solid tumours, and effect on autologous tumour cells; see text for more details. $8 \times 10^7$ cells from a spontaneously appearing adenocarcinoma ($adeno_{32}$) were fractionated for 150 min at 4°C. Aliquots of each fraction were tested at various concentrations for cytostasis at 48 h to $2 \times 10^3$ syngeneic embryo fibroblasts. The data in panel (a) indicate the cytostasis seen with 0·3% of the cells/fraction (representing a total unfractionated effector: target ratio of 120:1). In addition 1% of the cells in each fraction were added to each of 96-well Linbro plates. These cells were themselves used as cytostatic assay targets (b) for a pool of effector cells (sedimentation velocity 2–4 mm/h) from the solid tumour (×–×), normal spleen cells (●–●) or the tumour-bearer spleen cells (○–○). $2 \times 10^6$ effector cells were added per well in all cases.

female mice (4/group) to ensure ourgrowth of tumour on primary transplant from all fractions. Effector cells for assay of cytostasis on the fractionated tumour target cells were prepared from the small-cell region of the tumour-cell gradient (2–4 mm/h) or from similar size (same sedimentation velocity) spleen lymphocytes of the tumour bearer or normal mice. Control cultures, measuring incorporation of $[^3H]$-dT in the absence of added spleen lymphocytes, gave further evidence for adherent proliferating cells in all tumour-cell populations. These data are presented in the lower panel of Fig. 5. The variation in background $[^3H]$-dT incorporation in the fractions across the gradient was < 2·5-fold (data not shown). A correlation analysis of this background $[^3H]$-dT incorporation and the tumour volume of the primary transplants from each group (all mice developed palpable tumours by 40 days from s.c. inoculation) gave a $r = 0{\cdot}95$ (data not shown).

The first point apparent in these data, which are representative of 3 experiments of this type, is that only the small cells (sedimentation velocity 2–5 mm/h) derived from the tumour mass were able to cause a significant diminution in incorporation of $[^3H]$-dT by embryo fibroblasts (panel a). In contrast enhanced incorporation was seen when other fractions from the tumour mass were used in the assay; this may be caused by the tumour cells in the latter. More dramatic, however, were the effects when the different tumour-cell fractions were themselves the targets for the cytostasis assay (panel b). In this case marked heterogeneity in the activity of the same effector population (either normal spleen cells, ●—●, tumour-bearer spleen cells, ○—○, or small cytostatic cells from within the solid tumour, × — × ) was apparent, despite their having been tested at identical effector:target ratios for the 3 effector populations shown. Indeed tumour-bearer spleen lymphocytes were singularly ineffective against all but the slowest-sedimenting tumour-cell populations (peak sedimentation velocity 5–8 mm/h). Since tumour growth from the different fractions *in vivo* (s.c. implant) and background $[^3H]$-dT incorporation from the fractions *in vitro* were highly correlated (see above) the data of panel (b) may reflect a heterogeneity of target:effector cell interaction within a growing tumour mass, the understanding of which is of importance to our understanding of tumour metastasis.

### DISCUSSION

In an earlier paper (Gorczynski & MacRae, unpublished) we showed that a good correlation existed between those cells capable of demonstrating cytotoxicity to syngeneic embryo fibroblasts *in vitro* and those able to modify the growth of s.c. transplants of spontaneous adenocarcinoma cells. However, no such correlation between cytotoxic potential (*in vitro*) and the ability to modify lung nodule growth after i.v. transfer of tumour cells has been seen (manuscript in preparation). This may not be altogether surprising in view of the evidence that suitably activated macrophages may be a predominant factor in the regulation of metastatic tumour growth (Hibbs, 1973) whereas we have demonstrated that lymphocyte preparations are probably important for cytotoxic activity *in vitro* (Gorczynski, 1976*b*). Nevertheless, we feel a wealth of data points to the importance of immune reactions to embryonic antigens in natural anti-tumour immunity (Gorczynski, 1976*c*; Gorczynski & MacRae, unpublished; Baldwin *et al.*, 1974; Low & Appella, 1976; Castro *et al.*, 1973).

In an attempt to develop alternative assays for cells sensitized against embryo-associated determinants, we have explored an *in vitro* cytostasis assay which uses labelling of targets after culture (rather than pre-labelled targets). The data of Tables I and II show that this cytostatic assay can be used quantitatively to compare effector-cell populations. When this is done to assess the reactivity which appears spontaneously in cultures in normal mouse spleen lymphocytes it is apparent that cytotoxic and cytostatic activities are properties of independent cell populations. Abundant data exist in other tumour systems for a difference in effector-cell type according to the assay system used for detection of immunity (Parmiani & Lembo, 1974; Lamon *et al.*, 1972; Leclerc *et al.*, 1972; Owen & Seeger, 1972). Indeed (see Table II and Fig. 2) there is evidence that cytostasis itself is a property of different biological types of cells. Thus, one such cell pool is dependent upon activity for slow-sedimenting T cells (either adherent or requiring accessory adherent cells for activity) whilst another cell pool is represented by fast-sedimenting glass-adherent non-T cells. Further, this latter cell type apparently lacks embryonic-antigen specificity (though its induction may depend upon immune recognition of embryo-associated antigens; see Figs 4 and 5). These data are reminiscent of results with a virally induced tumour in mice (Owen & Seeger, 1973). Interestingly, whereas evidence for "memory" of exposure of embryo-associated antigens persisted in retired breeder mice, exposure to cross-reactive (with embryo antigens) tumour antigens by transplantation followed by surgical removal of tumour cells, led to a *reversible* increase in cytostatic activity (Fig. 4). This decline in cytostasis may be related to subsequent metastatic spread in these animals.

Recently, spontaneous cell-mediated cytotoxicity (detectable in short-term $^{51}$Cr-release tests) has been demonstrated in a number of species including man and mice (Herberman & Holden, 1978; Kiessling & Haller, 1978) and these effector (natural killer, NK) cells have been implicated in immune surveillance and tumour immunity (Haller *et al.*, 1977; Warner *et al.*, 1977). While it has been claimed that human and rat NK cells are Fc-receptor (for IgG) bearing subpopulations of T cells (Kay *et al.*, 1977) Kall & Koren (1978) have shown that the most active NK cells in humans are non-T cells which do not adhere to nylon-wool columns. There is no information available to date (see review by Kiessling & Wigzell, 1979) which would allow us to compare the specificity of NK cells with the cytostatic effector cells shown here, which (see Fig. 2 and Table I) recognize embryo-associated determinants and can be induced in culture by exposure to them.

In a more direct attempt to explore the role of cytostatic effector cells in autologous tumour immunity, however, we have investigated the presence of such cells within the growing tumour-cell mass (Fig. 5). There were two types of finding. Firstly, it was apparent that cells with this activity did indeed exist, though their activity did not correlate with previous analysis of regulation of s.c. tumour growth. Secondly, and more importantly, different fractions of cells isolated from within the solid tumour were found to differ in their capacity to be inhibited (from [$^3$H]-dT uptake) by cytostatic tumour-derived or, more specifically, tumour-bearer spleen-cell-derived effector cells. (Heterogeneity of *effector* cells in a virally induced mouse tumour, as judged by the ability of such cytotoxic effector cells to be blocked by tumour-associated antigens, was reported earlier by us (Gorczynski & Knight, 1975). These findings may implicate a role for a cytostatic assay in the assessment of metastatic capacity of various tumour cell populations, an hypothesis which is explored further in the following manuscript.

The authors would like to thank Ms Susan Oliphant for her excellent assistance and Dr G. Price for his many helpful suggestions in the preparation of this manuscript. This work was supported by the Canadian Medical Research Council (Grant No. MA-5440) and the National Cancer Institute of Canada.

### REFERENCES

ATTIA, M. A., DEOME, K. B. & WEISS, I.W. (1965) Immunology of spontaneous mammary carcinomas in mice. *Cancer Res.*, **25**, 451.

BALDWIN, R. W., EMBLETON, M., PRICE, M. R. & VOSE, B. M. (1974) Embryonic antigen expression on experimental rat tumors. *Transpl. Rev.*, **20**, 77.

BASOMBRIO, M. D. & PREHN, R. T. (1972) Search for common antigenicities between embryonic and tumoral tissues. *Medicina*, **32**, 42.

CASTRO, J. E., LANCE, E. M., MEDAWAR, P. B., ZANELLI, J. & HUNT, R. (1973) Foetal antigens and cancer. *Nature*, **243**, 225.

ECCLES, S. A. & ALEXANDER, P. (1974) Macrophage content of tumors in relation to metastatic spread and host immune reaction. *Nature*, **250**, 667.

FREUND, J. E. (1962) *Mathematical Statistics.* New York: Prentice-Hall.

GORCZYNSKI, R. M. (1976*a*) Autoreactivity developing spontaneously in cultured mouse spleen cells. I. Evidence that cytotoxicity is directed against embryo associated antigen. *Immunology*, **31**, 607.

GORCZYNSKI, R. M. (1976*b*) Autoreactivity developing spontaneously in cultured mouse spleen cells. II. Comparison of cytotoxicity of cultured male and female spleen cells. *Immunology*, **31**, 615.

GORCZYNSKI, R. M. (1976*c*) Autoreactivity developing spontaneously in cultured mouse spleen cells. III. Inhibition of anti-embryo cytotoxicity in male T lymphocytes by non-T cells. *Immunology*, **31**, 625.

GORCZYNSKI, R. M. (1976*d*) Control of the immune response: Role of macrophages in regulation of antibody- and cell-mediated immune responses. *Scand. J. Immunol.*, **5**, 1031.

GORCZYNSKI, R. M. (1978) Response of tumour-related and normal lymphocytes to antigens on fibroblasts from embryos of varying age. *Br. J. Cancer*, **37**, 786.

GORCZYNSKI, R. M. & KNIGHT, R. A. (1975) Immunity to murine sarcoma virus induced tumours. IV. Direct cellular cytolysis of $^{51}$Cr labelled target cells *in vitro* and analysis of blocking factors which modulate cytotoxicity. *Br. J. Cancer*, **31**, 387.

HALLER, O., HANSSON, M., KIESSLING, R. & WIGZELL, H. (1977) Role of non-conventional natural killer cells in resistance against syngeneic tumor cells *in vivo. Nature*, **270**, 609.

HERBERMAN, R. B. & HOLDEN, H. (1978) Natural cell-mediated immunity. In *Advances in Cancer Research.* Vol. 27. Eds Klein & Weinhouse. New York: Academic Press. p. 305.

HIBBS, J. B. (1973) Macrophage non-immunologic recognition: Target cell factors related to contact inhibition. *Science*, **180**, 868.

KALL, M. A. & KOREN, H. S. (1978) Heterogeneity of human natural killer cell populations. *Cell Immunol.*, **40**, 58.

KAY, H. D., BONNARD, G. D., WEST, W. H. & HERBERMAN, R. B. (1977) A functional comparison of human Fc-receptor-bearing lymphocytes active in natural cytotoxicity and antibody-dependent cellular cytotoxicity. *J. Immunol.*, **118** 2058.

KIESSLING, R. & HALLER, O. (1978) Natural killer cells in the mouse: An alternative immune surveillance mechanism? *Contemp. Top. Immunobiol.*, **8**, 171.

KIESSLING, R. & WIGZELL, H. (1979) An analysis of the murine NK cells as to structure function and biological relevance. *Immunol. Rev.*, **44**, 165.

LAMON, E. W., SKURZAK, H. M., KLEIN, E. & WIGZELL, H. (1972) *In vitro* cytotoxicity by a non-thymus-processed lymphocyte population with specificity for a virally determined tumor cell surface antigen. *J. Exp. Med.*, **136**, 1072.

LAW, L. W. & APPELLA, E. (1976) Immunogenic properties of solubilized tumor antigen from an RNA virus-transformed neoplasm. *Nature*, **243**, 83.

LECLERC, L. C., GOMARD, E. & LEVY, J. P. (1972) Cell-mediated reaction against tumors induced by oncornaviruses. I. Kinetics and specificity of the immune response in murine sarcoma virus (MSV) —induced tumors and transplanted lymphomas. *Int. J. Cancer*, **10**, 589.

OWEN, J. J. T. & SEEGER, R. C. (1973) Immunity to tumours of the murine leukaemia–sarcoma virus complex. *Br. J. Cancer*, **28**, 26, Suppl. I,

PARMIANI, G. & LEMBO, R. (1974) Effect of anti-embryo immunization on methylcholanthrene induced sarcoma growth in BALB/c mice. *Int. J. Cancer*, **14**, 555.

PEARSON, G. & FREEMAN, G. (1968) Evidence suggesting a relationship between polyoma virus induced transplantation antigen and normal embryonic antigen. *Cancer Res.*, **28**, 1665.

RUSSELL, S. W., GILLESPIE, G. Y., HANSEN, C. G. & COCHRANE, C. G. (1976) Inflammatory cells in solid murine neoplasms. II. Cell types found throughout the course of Moloney sarcoma regression or progression. *Int. J. Cancer*, **18**, 331.

TING, C. C. (1968) Failure to induce transplantation resistance against polyoma tumor cells to syngeneic embryonic tissues. *Nature*, **217**, 858.

WARNER, N. L., WOODRUFF, M. F. A. & BURTON, R. C. (1977) Inhibition of the growth of lymphoid tissue in syngeneic athymic (nude) mice. *Int. J. Cancer*, **20**, 146.